# Continuous low-dose antibiotic prophylaxis for adults with repeated urinary tract infections (AnTIC): a randomised, open-label trial

**DOI:** 10.1016/S1473-3099(18)30279-2

**Published:** 2018-09

**Authors:** Holly Fisher, Yemi Oluboyede, Thomas Chadwick, Mohamed Abdel-Fattah, Catherine Brennand, Mandy Fader, Simon Harrison, Paul Hilton, James Larcombe, Paul Little, Doreen McClurg, Elaine McColl, James N'Dow, Laura Ternent, Nikesh Thiruchelvam, Anthony Timoney, Luke Vale, Katherine Walton, Alexander von Wilamowitz-Moellendorff, Jennifer Wilkinson, Ruth Wood, Robert Pickard

**Affiliations:** a Institute of Health and Society, Newcastle University, Newcastle upon Tyne, UK; b Newcastle Clinical Trials Unit, Newcastle University, Newcastle upon Tyne, UK; c Institute of Cellular Medicine, Newcastle University, Newcastle upon Tyne, UK; d Institute of Applied Health Sciences, University of Aberdeen, Aberdeen, UK; e Health Sciences, University of Southampton, Southampton, UK; f Department of Urology, Mid-Yorkshire Hospitals NHS Trust, UK; g Skerne Medical Centre, Sedgefield, UK; h Nursing, Midwifery and Allied Health Professions Research Unit, Glasgow Caledonian University, Glasgow, UK; i Department of Urology, Cambridge University Hospitals NHS Foundation Trust, Cambridge, UK; j Bristol Urological Institute, North Bristol NHS Trust, Bristol, UK; k Department of Microbiology, Newcastle upon Tyne Hospitals NHS Foundation Trust, Newcastle upon Tyne, UK

## Abstract

**Background:**

Repeated symptomatic urinary tract infections (UTIs) affect 25% of people who use clean intermittent self-catheterisation (CISC) to empty their bladder. We aimed to determine the benefits, harms, and cost-effectiveness of continuous low-dose antibiotic prophylaxis for prevention of recurrent UTIs in adult users of CISC.

**Methods:**

In this randomised, open-label, superiority trial, we enrolled participants from 51 UK National Health Service organisations. These participants were community-dwelling (as opposed to hospital inpatient) users of CISC with recurrent UTIs. We randomly allocated participants (1:1) to receive either antibiotic prophylaxis once daily (prophylaxis group) or no prophylaxis (control group) for 12 months by use of an internet-based system with permuted blocks of variable length. Trial and laboratory staff who assessed outcomes were masked to allocation but participants were aware of their treatment group. The primary outcome was the incidence of symptomatic, antibiotic-treated UTIs over 12 months. Participants who completed at least 6 months of follow-up were assumed to provide a reliable estimate of UTI incidence and were included in the analysis of the primary outcome. Change in antimicrobial resistance of urinary and faecal bacteria was monitored as a secondary outcome. The AnTIC trial is registered at ISRCTN, number 67145101; and EudraCT, number 2013-002556-32.

**Findings:**

Between Nov 25, 2013, and Jan 29, 2016, we screened 1743 adult users of CISC for eligibility, of whom 404 (23%) participants were enrolled between Nov 26, 2013, and Jan 31, 2016. Of these 404 participants, 203 (50%) were allocated to receive prophylaxis and 201 (50%) to receive no prophylaxis. 1339 participants were excluded before randomisation. The primary analysis included 181 (89%) adults allocated to the prophylaxis group and 180 (90%) adults in the no prophylaxis (control) group. 22 participants in the prophylaxis group and 21 participants in the control group were not included in the primary analysis because they were missing follow-up data before 6 months. The incidence of symptomatic antibiotic-treated UTIs over 12 months was 1·3 cases per person-year (95% CI 1·1–1·6) in the prophylaxis group and 2·6 (2·3–2·9) in the control group, giving an incidence rate ratio of 0·52 (0·44–0·61; p<0·0001), indicating a 48% reduction in UTI frequency after treatment with prophylaxis. Use of prophylaxis was well tolerated: we recorded 22 minor adverse events in the prophylaxis group related to antibiotic prophylaxis during the study, predominantly gastrointestinal disturbance (six participants), skin rash (six participants), and candidal infection (four participants). However, resistance against the antibiotics used for UTI treatment was more frequent in urinary isolates from the prophylaxis group than in those from the control group at 9–12 months of trial participation (nitrofurantoin 12 [24%] of 51 participants from the prophylaxis group *vs* six [9%] of 64 participants from the control group with at least one isolate; p=0·038), trimethoprim (34 [67%] of 51 *vs* 21 [33%] of 64; p=0·0003), and co-trimoxazole (26 [53%] of 49 *vs* 15 [24%] of 62; p=0·002).

**Interpretation:**

Continuous antibiotic prophylaxis is effective in reducing UTI frequency in CISC users with recurrent UTIs, and it is well tolerated in these individuals. However, increased resistance of urinary bacteria is a concern that requires surveillance if prophylaxis is started.

**Funding:**

UK National Institute for Health Research.

## Introduction

Cohort studies from Europe and North America show that people who use clean intermittent self-catheterisation (CISC) to empty their bladders, possibly due to neurological disease such as multiple sclerosis or failure of urinary sphincter relaxation, have an average prevalence of repeated symptomatic urinary tract infections (UTIs) of 25%.^1^ Factors that could increase the risk of UTIs include being female, having neurological bladder dysfunction, and having bacterial colonisation of the urine.^2^ Prevention of UTIs with continuous low-dose antibiotic prophylaxis has been tested in five previous trials involving 363 participants that were summarised in a Cochrane review.^3^ Results were inconsistent, showing no difference in three trials and a benefit in two trials. We repeated this Cochrane search of the medical literature with a later end date of November, 2017, but we did not identify any further reports.

**Research in context**

**Evidence before this study**

People who need to perform clean intermittent self-catheterisation (CISC) to empty their dysfunctional urinary bladders are known to be at high risk of repeated symptomatic urinary tract infections (UTIs); an average prevalence of UTIs of 25% in these people has been estimated in European and North American cohort studies. These infections exacerbate their existing poor health. One possible intervention to reduce UTI frequency is the use of continuous low-dose antibiotic prophylaxis, which appears to be effective for women with normal functioning bladders who have repeated UTIs. A Cochrane systematic literature review up to September, 2011, identified five small trials that enrolled specific groups of CISC users. The three trials that involved children and the two trials that involved patients with recent spinal injuries showed inconsistent findings for UTI prevention and provided limited evidence of effectiveness of continuous antibiotic prophylaxis. We did an updated search from August, 2011, to November, 2017, and identified no further relevant trials. As in the Cochrane review, we searched CENTRAL, MEDLINE, CINAHL, ClinicalTrials.gov, WHO International Clinical Trials Registry Program, and the UK Clinical Research Network for papers published between Sept 18, 2011, and Nov 30, 2017. The search terms used were “(design.rct* or design.cct*) AND ({intvent.mech.cath*}”, “{intvent.mech.device*}”, “{intvent.mech.sheaths.}”, “{intvent.prevent.antibiotics*}”, “{intvent.prevent.antinfect.*}”, “{intvent.prevent.cath*}”, “{intvent.prevent.cleaning fluids*}”, “{intvent.prevent.surg*}”, “{intvent.surg.intraoperativemanagement*}”, “{intvent.surg.postsurgman*}”, “{intvent.surg.presurgman*.}”, and “{intvent.surg.urethrotomy.})”. We used no language restrictions in our search.

**Added value of this study**

We provide clear evidence from a robustly planned and conducted randomised controlled trial that antibiotic prophylaxis is effective and potentially efficient for treatment of a wider population of adults who use CISC and have repeated UTIs, regardless of the cause of incomplete bladder emptying. The results give a precise estimate of the degree of benefit that this patient group can expect when they use this intervention. Additionally, we quantify the main drawback of this treatment: increased antimicrobial resistance of the bacteria that colonise urine, urinary pathogens, and the faecal microbiome.

**Implications of all the available evidence**

The results of our trial reflect similar findings in children to show that adult users of CISC with repeated UTIs, irrespective of the underlying cause of their bladder dysfunction and the presence of other risk factors for UTIs, are likely to benefit from use of antibiotic prophylaxis through reduced frequency of UTIs during 12 months of use. The disadvantage of this approach is that antimicrobial resistance of potential and active pathogens is likely to increase. The long-term implications of this intervention are uncertain, but increased pathogen resistance might make it more difficult to treat established infections in individuals, and increased resistance of bacteria that colonise urine and contribute to the faecal microbiome are a public health concern. The severity of individual patient distress from repeated UTIs and local threats from antimicrobial resistance should simultaneously be considered when appraising and implementing this evidence of benefit of treatment.

The main potential harm of prophylaxis is the development of antimicrobial resistance by urinary pathogens, which would make it more difficult to treat infections and would be a public health concern.^4^ Resistance to antibiotics was examined in two of the trials of the Cochrane review, but this review found no difference in antimicrobial resistance between prophylaxis and placebo groups in adults who were admitted to hospital with spinal injury.^5^ Increased antimicrobial resistance was found in urinary isolates from children with neural tube defects who were receiving prophylaxis compared with those who had stopped routine prophylaxis.^6^, ^7^

Data^8^ from National Health Service (NHS) England showed that 71 million CISC catheters were prescribed for adult use in 2016. Assuming a use of four catheters per person per day,^9^ these data imply a prevalence of 74 people who use catheters per 100 000 people, which is similar to the prevalence observed in France (62 users per 100 000 people).^10^ Although precise data are unavailable, the prevalence of repeated UTIs that is associated with CISC use is likely to be similar in other countries with sufficient health-care resources.

We aimed to determine the clinical effectiveness and cost-effectiveness of continuous low-dose antibiotic prophylaxis in reducing the frequency of UTIs over 12 months in adult users of CISC.

## Methods

### Study design and participants

We did a randomised, open-label, parallel group, superiority trial, in which we recruited participants from 51 NHS organisations (hospitals, clinics, and community care providers) and treated them in the community. Participants self-medicated at home.

Local research staff at sites screened established adult users of CISC who were anticipated to continue CISC use for at least 12 months for participation in the trial. For study inclusion, participants had to have had either at least two episodes of symptomatic UTIs that were related to CISC within the past 12 months or at least one episode of UTI requiring hospital admission. Participants were excluded if they were unable to tolerate all three of the agents used for UTI prophylaxis in the trial and, if they were women, if they were intending to become pregnant or were pregnant or breastfeeding.

Participants were identified from health records and clinic visits and an outline of the trial was explained to them. Those who were interested in study participation were seen by local researchers, who discussed the trial and provided written information, and those who decided to participate provided written consent for the 12-month trial period and, if willing, consent for additional follow-up at 18 months. They provided data (on demongraphics, CISC use, UTIs, and cause of lower urinary tract dysfunction) at baseline and were randomly allocated to groups at this stage. Participants who had given written informed consent and who were already taking antibiotic prophylaxis against UTIs were asked to stop and complete a 3-month period without prophylaxis before baseline assessment and randomisation. Ethical approval was given on Aug 1, 2013, by the NHS Research Ethics Service Committee North East (Sunderland; 13/NE/0196). The trial protocol has been published.^11^

### Randomisation and masking

Participants were randomly allocated (1:1) to receive either antibiotic prophylaxis (experimental) or no prophylaxis (control). Randomisation was done centrally by an internet-based system that used permuted random blocks of variable length (two, four, and six). A statistician that was not otherwise involved with the study produced the final allocation schedule, including stratification by three variables: previous frequency of UTIs (less than four episodes per year *vs* at least four episodes per year), a diagnosis of neurological dysfunction of the lower urinary tract, and sex. Clinical trial unit staff and central laboratory staff who were assessing outcomes were masked to allocation. By necessity, participants, treating clinicians, and local research staff were masked to block size but not to allocation. For those allocated to prophylaxis, the clinician started the patient on a study drug for UTI prophylaxis that was suitable to the individual. The drugs given were 50 mg nitrofurantoin, 100 mg trimethoprim, or 250 mg cefalexin. These drugs were prescribed to be taken once daily and were supplied by the standard NHS mechanisms and manufacturers licensed by the UK Medicines and Healthcare Products Regulatory Agency.

### Procedures

Participants were scheduled to be reviewed in person or by telephone at 1, 3, 6, 9, and 12 months after randomisation by local research staff. At the 1-month review, we checked their tolerance of trial medication and their understanding of trial assessments. If necessary, an alternative antibiotic was substituted. At 3, 6, 9, and 12 months, participants completed trial questionnaires and research staff completed a case report form. These forms collected outcome measures, adverse effects, and adherence to their allocated treatment. Additionally, participants submitted a specimen of urine that was taken during an asymptomatic period at the baseline and at 3, 6, 9, and 12 months. Participants also submitted a perianal swab at baseline and at 6 and 12 months, which they posted to the central trial laboratory (Department of Microbiology, Freeman Hospital, The Newcastle upon Tyne Hospitals NHS Foundation Trust, Newcastle upon Tyne, UK) in supplied, approved packaging. If participants had a UTI for which they had sought antibiotic treatment, they submitted a UTI report form to the central trial office and posted a urine specimen in a sterile universal containing 18 g/L boric acid (International Scientific Supplies, Bradford, UK) to the trial laboratory before starting antibiotic treatment.

At the end of the trial, participants were asked to discuss whether to continue, stop, or start prophylaxis with their clinician. Participants who had consented to further follow-up were sent a questionnaire regarding antibiotic use at 18 months after randomisation, and they were asked to post a further urine specimen that was taken while they were asymptomatic and a perianal swab to the trial laboratory.

### Outcomes

The primary outcome was incidence of symptomatic, antibiotic-treated UTIs during the 12 months of trial participation. These UTIs were defined as the presence of at least one symptom from a prespecified list, including urinary symptoms, change in urine appearance, abdominal pain, difficulty in catheterisation, systemic infective symptoms, or increased limb spasticity (appendix), and the participant had to be taking a treatment course of antibiotics for their UTI. The results of the primary outcome were collected by participant completion of a UTI report form and of the 3-monthly trial questionnaire and completion of the 3-monthly case report form by local research staff. The three sources of data were centrally assessed by two members of the trial team (CB and AvW-M) who did not know the allocated group of each participant. The assessment was done with the protocol to decide attribution of weighting to give each datapoint. A third member (RP) arbitrated over discrepancies.

Secondary microbiological outcomes were the incidence of microbiologically confirmed symptomatic antibiotic-treated UTIs; incidence of asymptomatic bacteriuria; and change in the frequency of antimicrobial resistance of bacteria that were isolated from urine samples during UTIs and during asymptomatic periods and of *Escherichia coli* isolated from perianal swabs. Microbiological confirmation of UTIs was defined as a significant positive culture from a urine sample that was posted to the central laboratory at the time of a reported UTI or, if no sample was received, a positive culture reported by a local laboratory. Asymptomatic bacteriuria was defined as a positive culture from urine specimens that were received by the central laboratory that were sent at 3-monthly intervals in asymptomatic periods. We only assessed antimicrobial resistance of bacteria that were isolated from urine during UTIs and asymptomatic periods and of *E coli* isolated from perianal swabs in specimens that were submitted to the central laboratory. The central laboratory was accredited to ISO15189 (the UK representative of the International Organization for Standardization) and analyses were done in accordance with standards set by Public Health England and the European Committee on Antimicrobial Susceptibility Testing.^12^ A significant positive urine culture was defined as the presence of up to two isolates of at least 1 × 10^4^ colony forming units per mL.^13^

Secondary clinical outcomes included the incidence of febrile UTIs over 12 months; hospital admission due to UTIs over 12 months; overall satisfaction with allocated treatment strategy, which was assessed by the Treatment Satisfaction Questionnaire for Medication (TSQM)^14^ at 12 months; the effect on health status, assessed by participant completion of the Medical Outcomes Short Form-36 item questionnaire (SF-36; version 2, 1-week recall version)^15^ at baseline, 6 and 12 months, and at the time of each UTI; kidney function, determined by change in estimated glomerular filtration rate, and liver function, determined by alanine transaminase concentration, from baseline to 12 months; and incidence of adverse events associated with prophylactic and treatment antibiotics over 12 months.

Cost-effectiveness from a health-care perspective was primarily assessed as the incremental cost per UTI avoided. Health-care costs were collected through 3-monthly case report forms, which were completed by local research staff, and from a participant-completed health use questionnaire at 6 and 12 months. Additionally, we did cost-utility analyses to assess the incremental cost per quality-adjusted life-years (QALY) gained using participant responses to the SF-36 questionnaire, which was completed at baseline, at 6 and 12 months, and at the time of a UTI. For the incremental cost per QALY estimates, utility values that indicated perceived health status between 0 (death) and 1 (perfect health) were calculated using an established mapping algorithm to the SF-6D.^16^ Finally, a cost-benefit analysis was done by use of a bespoke questionnaire regarding their willingness-to-pay to avoid UTIs, which was administered to participants after completion of the 12-month study period.

### Statistical analysis

We considered a 20% reduction in average UTI frequency from three to 2·4 episodes per year to represent the minimal clinically important difference. Use of the Poisson rate test (for the primary analysis) required 158 participants in each group (316 in total) to complete the study, giving 90% power at the 5% significance level for detection of superiority of prophylaxis over no prophylaxis, which we increased to 372 to allow 15% attrition.

The primary measure of effect was the relative difference in the incidence of symptomatic, antibiotic-treated UTIs between the two groups during the 12-month observation period. All participants who completed at least 6 months of follow-up were included in the modified intention-to-treat analysis, to report the comparative UTI incidence between groups, which we calculated as the incidence rate ratio (IRR) to allow for differing durations of follow-up.

The incidence of resistance over time to oral antibiotics that are commonly used against UTIs—amoxicillin, cefalexin, ciprofloxacin, co-trimoxazole, co-amoxiclav, mecillinam, nitrofurantoin, and trimethoprim—were summarised graphically by group. A χ^2^ test was used to compare bacterial resistance rates between prophylaxis and control groups by examination of all isolates from surveillance urine specimens collected between 9 and 12 months and in strains of *E coli* isolated from perianal swabs between 6 and 12 months. Tests for trend of change in antimicrobial resistance were done separately for each group for isolates from surveillance urine.

Univariate analysis of change in kidney and liver function was done with a two-sample *t* test with additional analysis of covariance that used the covariates identified during the primary outcome modelling.

Prespecified additional analyses of the primary outcome were done to check the robustness of the primary result. These included not counting days participants spent taking treatment antibiotics for UTIs in the exposure time. Additional prespecified analyses associated with the primary outcome were done by the addition of an interaction term to the model, to explore subgroup effects (less than four *vs* at least four episodes per year) of UTIs at baseline. Additional modelling of the primary analysis adjusted the IRR for the effects of covariates, including stratification factors and other possible risk factors for UTIs (age, functional cause of poor bladder emptying, type of catheter, daily frequency of CISC use, use of prophylaxis in previous 12 months, kidney dysfunction, and presence of asymptomatic bacteriuria at baseline), in a manner analogous to the primary analysis. We also did a sensitivity analysis of the primary outcome by use of negative binomial regression and a strict intention-to-treat definition (appendix). Analyses were done in STATA version 14. The study was overseen by independent trial steering and data monitoring committees. The AnTIC trial is registered at ISRCTN, number 67145101; and EudraCT, number 2013-002556-32.

### Role of the funding source

The funder of the study had no role in study design, data collection, data analysis, data interpretation, or writing of the report. The corresponding author had full access to all the data in the study and had final responsibility for the decision to submit for publication.

## Results

**Figure 1:**
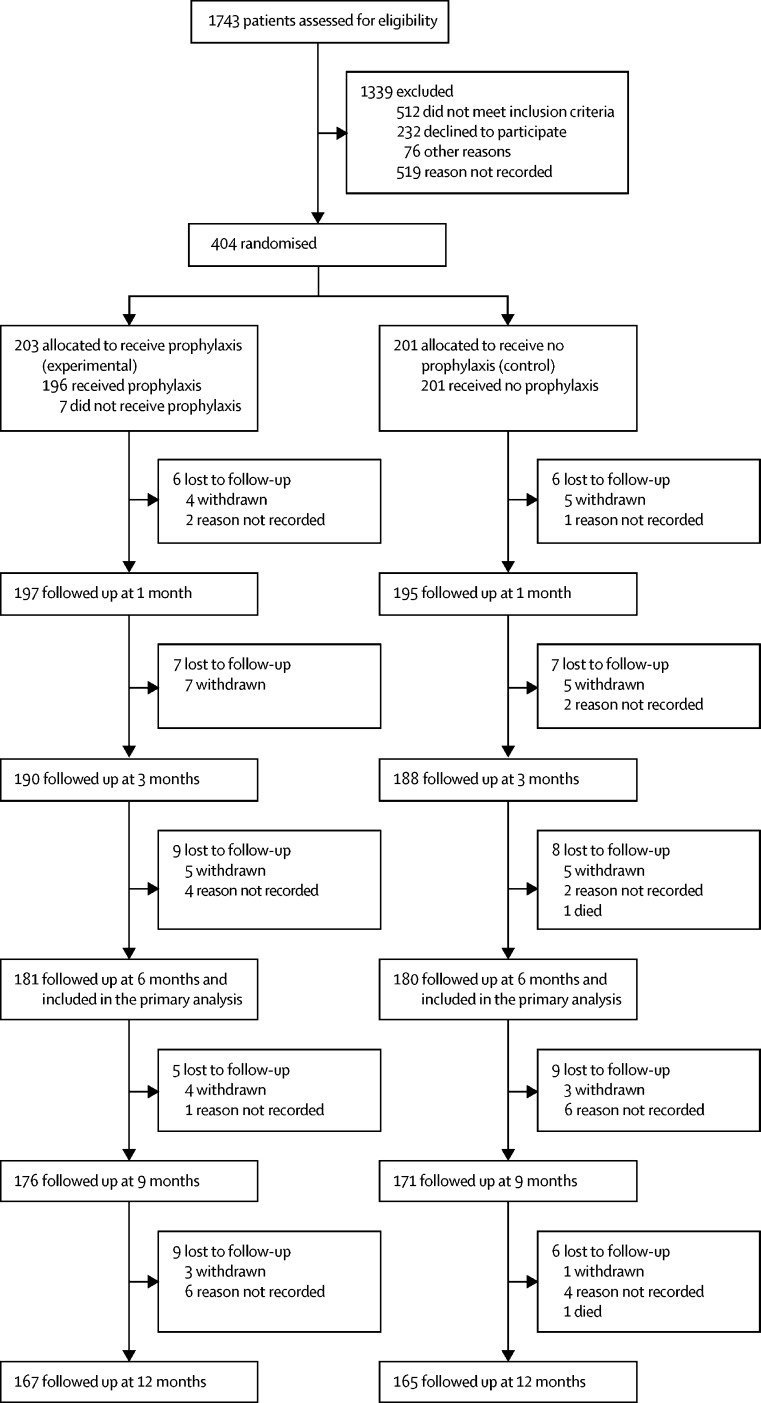
Trial profile

Between Nov 25, 2013, and Jan 29, 2016, we screened 1743 patients for inclusion in the trial. Between Nov 26, 2013, and Jan 29, 2016, 404 participants were randomly allocated to groups: 203 (50%) participants were allocated to the prophylaxis group and 201 (50%) to the no prophylaxis (control) group (figure 1). 1339 participants were excluded before randomisation. We included 361 (89%) participants in the primary analysis, comprising 181 (89%) participants from the prophylaxis group and 180 (90%) participants from the control group. 22 participants in the prophylaxis group and 21 participants in the control group were not included in the primary analysis because they were missing follow-up data before 6 months. 34 (17%) participants in the prophylaxis group stopped taking antibiotic prophylaxis during the 12-month observation period and 26 (13%) participants allocated to receive no prophylaxis started antibiotic prophylaxis during the 12-month observation period. Of the 34 who stopped prophylaxis, 23 (68%) were in the first 6 months. Of the 26 who started prophylaxis in the no prophylaxis group, 17 (65%) were in the first 6 months. Participants who were given the treatment of the other group were included in the primary analysis, if otherwise eligible, and they were evaluated by their original allocation. The most common reasons for patient withdrawal during the first 12 months were unwillingness to continue with the study, unwillingness to continue as a result of comorbidities, and study burden being too great. Participant characteristics were similar between the groups at baseline (table 1). At 12 months, 77 (78%) of 99 participants allocated to prophylaxis who expressed a preference stated that they wished to continue to receive prophylaxis; in the control group, 83 (80%) of 104 participants stated that they wished to continue without prophylaxis.

**Table 1:** Baseline characteristics

		**Prophylaxis group (n=203)**	**No prophylaxis group (n=201)**
Age, years		59·1 (17·0)	60·1 (15·6)
Weight, kg		78·9 (17·4)	81·3 (16·2)
Sex			
	Male	115 (57%)	114 (57%)
	Female	88 (43%)	87 (43%)
Number of urinary tract infections in 12 months before randomisation			
	<4	71 (35%)	78 (39%)
	≥4	132 (65%)	123 (61%)
Cause of bladder dysfunction			
	Neurological	80 (39%)	78 (39%)
	Non-neurological	123 (61%)	123 (61%)
Creatinine clearance, mL/min		89·8 (68·6–121·4)	99·1 (71·9–124·2)
Type of clean intermittent catheterisation			
	By self	201 (99%)	198 (99%)
	By spouse or carer	1 (<1%)	2 (1%)
	Missing data	1 (<1%)	1 (<1%)
Planned future duration of need for clean intermittent catheterisation			
	Between 1 and 2 years	0	4 (2%)
	Between 2 and 5 years	0	1 (<1%)
	Indefinite	182 (90%)	181 (90%)
	Not known	20 (10%)	14 (7%)
	Missing data	1 (<1%)	1 (<1%)
Route of clean intermittent catheterisation			
	Urethra	196 (97%)	195 (97%)
	Mitrofanoff catheterisable stoma	6 (3%)	5 (2%)
	Missing data	1 (<1%)	1 (<1%)
Type of catheter used			
	Single use	200 (99%)	199 (99%)
	Reusable	2 (1%)	2 (1%)
	Missing data	1 (<1%)	0
Hydrophilic-coated catheter used?			
	Yes	189 (93%)	192 (96%)
	No	9 (4%)	8 (4%)
	Missing data	5 (2%)	1 (<1%)
Frequency of clean intermittent self-catheterisation over 24 h		3·8 (2·2)	4·1 (2·9)
Main functional reason for requiring clean intermittent catheterisation			
	Bladder failure (or underactivity)	139 (68%)	128 (64%)
	Bladder outlet obstruction	49 (24%)	56 (28%)
	Bladder augmentation or replacement	13 (6%)	16 (8%)
	Missing data	2 (1%)	1 (<1%)
Urinary tract infection details			
	Number of infections in the 12 months before randomisation reported by the patient	4·0 (3·0–6·0)	4·0 (3·0–7·0)
	Positive urine culture reports in the 12 months before randomisation	2·0 (1·0–4·0)	2·0 (1·0–4·0)
	Number of months of antibiotic prophylaxis for urinary tract infections in 12 months before randomisation	0·0 (0·0–1·0)	0·0 (0·0–1·0)
Results of central laboratory culture of urine at baseline			
	Negative	93 (46%)	84 (42%)
	Positive	76 (37%)	77 (38%)
	Missing data	34 (17%)	40 (20%)

Data are n (%), median (IQR), or mean (SD).

During the 12-month study, the incidence of symptomatic antibiotic-treated UTIs in the prophylaxis group was 1·3 cases per person-year (95% CI 1·1–1·6) and 2·6 cases per person-year (2·3–2·9) for no prophylaxis (table 2). The IRR, which accounted for differing durations of follow-up, was 0·52 (95% CI 0·44–0·61; p<0·0001) in favour of prophylaxis, which indicated a 48% reduction in the incidence of UTIs associated with prophylaxis treatment. The median number of symptomatic, antibiotic-treated UTIs observed over 12 months was 1 (IQR 0–2) in the prophylaxis group and 2 (1–4) in the control group. In the prophylaxis group, 13 (10%) of 129 UTI reports stated that previously prescribed antibiotics (self-start therapy) were used for treatment compared with 57 (19%) of 299 reports in the no prophylaxis group.

**Table 2:** Incidence rates and incidence rate ratios of the primary and secondary outcomes, compared between the prophylaxis and control (no prophylaxis) groups

	Prophylaxis group (n=181)*	No prophylaxis group (n=180)*	**Incidence rate ratio (95% CI)**	**p value**
**Symptomatic, antibiotic-treated urinary tract infections**†				
All eligible participants	1·3 (1·1–1·6)	2·6 (2·3–2·9)	0·52 (0·44–0·61)	<0·0001
<4 infections at baseline	0·8 (0·6–1·1)	1·7 (1·4–2·2)	0·46 (0·34–0·64)	0·45‡
≥4 infections at baseline	1·7 (1·3–2·0)	3·1 (2·7–3·6)	0·54 (0·45–0·64)	..
**Microbiologically confirmed urinary tract infections**§				
All eligible participants	0·74 (0·58–0·94)	1·5 (1·3–1·8)	0·49 (0·39–0·60)	<0·0001
<4 infections at baseline	0·32 (0·18–0·57)	1·2 (0·9–1·5)	0·28 (0·18,0·45)	0·01‡
≥4 infections at baseline	0·99 (0·77–1·3)	1·7 (1·4–2·1)	0·57 (0·45–0·72)	..
**Febrile urinary tract infections**§				
All eligible participants	0·11 (0·06–0·21)	0·16 (0·10–0·25)	0·71 (0·40–1·26)	0·24
<4 infections at baseline	0·07 (0·03–0·17)	0·12 (0·06–0·23)	0·62 (0·20–1·90)	0·79‡
≥4 infections at baseline	0·14 (0·06–0·30)	0·19 (0·11–0·32)	0·74 (0·38–1·45)	..
**Asymptomatic bacteriuria**§				
All eligible participants	1·4 (1·2–1·6)	1·6 (1·4–1·9)	0·88 (0·74–1·04)	0·14
<4 infections at baseline	1·5 (1·2–2·0)	2·0 (1·6–2·5)	0·77 (0·60–1·00)	0·18‡
≥4 infections at baseline	1·3 (1·1–1·6)	1·4 (1·1–1·6)	0·98 (0·77–1·23)	..

* Data are incidence rate (95% CI).

† Primary outcome.

‡ For interaction between subgroups (<4 and ≥4 infections at baseline) and treatment group.

§ Secondary outcome.

The microbiologically confirmed incidence of UTIs was 0·74 cases per person-year (95% CI 0·58–0·94) in the prophylaxis group and 1·5 cases per person-year (1·3–1·8) in the no prophylaxis group, giving an IRR of 0·49 (0·39–0·60) in favour of prophylaxis (table 2). 110 (61%) of 181 participants in the prophylaxis group and 113 (63%) of 180 participants in the control group had at least one positive 3-monthly surveillance urine culture that indicated a period of asymptomatic bacteriuria, and the incidence of asymptomatic bacteriuria did not differ between groups.

**Figure 2:**
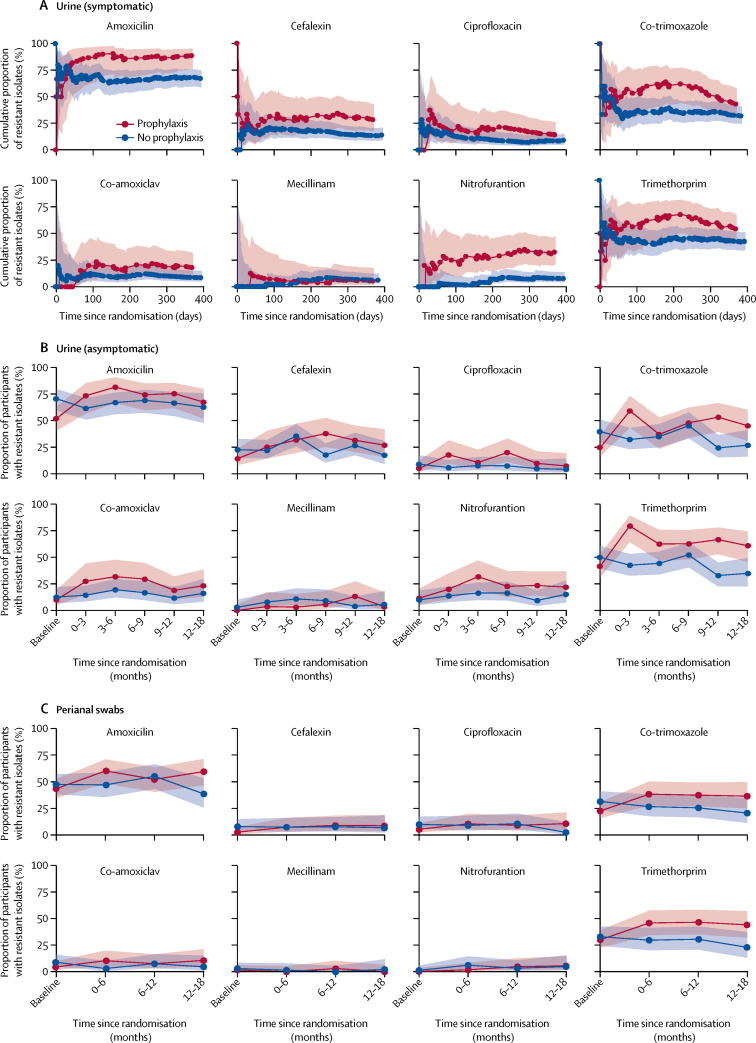
Antimicrobial resistance of isolates submitted by the prophylaxis group compared with the no prophylaxis group (A) Cumulative proportion (95% CI) of resistant isolates from urine specimens submitted during symptomatic urinary tract infections, plotted over the 12-month trial duration; (B) Incidence of resistance (95% CI) of bacteria isolated from urine specimens submitted during asymptomatic periods every 3 months over the 12-month trial duration and at an additional 18 month follow-up; and (C) Incidence of resistance (95% CI) of *Escherichia coli* isolated from perianal swabs that were submitted every 6 months over the 12-month trial duration and at an additional 18-month follow-up.

At baseline, the groups did not differ in frequency of antimicrobial resistance of urinary isolates to eight oral antibiotics that are commonly used for UTI treatment. During the 12-month trial, resistance appeared more common in isolates that were cultured from urine submitted during symptomatic UTIs by participants in the prophylaxis group than those in the control group (figure 2). In urine samples submitted during asymptomatic periods between months 9 and 12 of the trial, resistance to nitrofurantoin (12 [24%] of 51 participants with at least one isolate from the prophylaxis group *vs* six [9%] of 64 participants with at least one isolate from the control group; p=0·038), trimethoprim (34 [67%] of 51 participants *vs* 21 [33%] of 64 participants; p=0·0003), and co-trimoxazole (26 [53%] of 49 participants *vs* 15 [24%] of 62 participants; p=0·002) was significantly more frequent in the prophylaxis group than the control group. Over baseline and subsequent 3-monthly intervals during the 12-month trial period, resistance to amoxicillin (38 [52%] of 73 participants, 25 [74%] of 34 participants, 31 [82%] of 38 participants, 32 [74%] of 43 participants, and 37 [76%] of 49 participants; p=0·004), cephalexin (11 [14%] of 78 participants, ten [25%] of 40 participants, 13 [32%] of 41 participants, 17 [38%] of 45 participants, and 16 [31%] of 51 participants; p=0·005), co-trimoxazole (18 [25%] of 73 participants, 23 [59%] of 39 participants, 14 [37%] of 38 participants, 21 [48%] of 44 participants, 26 [53%] of 49 participants; p=0·006), and trimethoprim (32 [42%] of 77 participants, 31 [79%] of 39 participants, 25 [63%] of 40 participants, 27 [63%] of 43 participants, 34 [67%] of 51 participants; p=0·016) also significantly increased in isolates from urine specimens that were submitted during asymptomatic periods by participants in the prophylaxis group. There was no evidence of increasing resistance over time in the control group. Resistance of urinary isolates that were submitted during asymptomatic periods appeared to decrease at 18 months. There was no evidence that *E coli* isolated from perianal swabs of participants in the prophylaxis group had significantly more frequent resistance against any of the eight antibiotics tested than did participants in the control group at 6–12 months of the trial.

15 participants in the prophylaxis group (incidence 0·11 cases per person-year [95% CI 0·06–0·21]) and 22 in the control group (0·16 cases per person-year [0·10–0·25]) had at least one febrile UTI, giving an IRR of 0·71 (95% CI 0·40–1·26; p=0·24; table 2). Six participants in the prophylaxis group and eight participants in the control group were admitted to hospital due to UTIs. With the TSQM questionnaire, participants allocated to prophylaxis self-rated as being satisfied with their treatment (appendix) giving a mean overall score of 73·8 (SD 25·4; the range of scores for the TSMQ is 0–100). Health status, which was measured by participant completion of the SF-36 at 6 and 12 months, showed a small but significant improvement in the mental component score at 6 months but not at 12 months for participants using prophylaxis compared with no prophylaxis. Physical component scores did not differ between groups at 6 or 12 months. Responses to the SF-36 (which was completed by participants during UTIs) were used to generate utility values,^16^ which showed a small, non-significant decrease in both groups compared with baseline (appendix). There was no deterioration in kidney or liver function in either group (appendix).

Economic evaluation suggested that, on average, a strategy of antibiotic prophylaxis was more effective and more costly than no prophylaxis. The incremental cost was £99 per UTI avoided (table 3). There was a 60% chance that prophylaxis would be cost-effective should society be willing to pay £200 to avoid a UTI. The incremental cost per QALY over 12 months was £5059 (as determined by the cost-utility analysis). In the probabilistic analysis, the likelihood that use of prophylaxis would be considered cost-effective at a threshold value of £20 000 per QALY was 64%. Adjustment for utility values that were recorded at the time of UTI gave similar results. A probabilistic sensitivity evaluation from the willingness to pay analysis suggested that use of prophylaxis has a 66% chance of being considered more efficient and would give a higher net benefit than a no prophylaxis strategy if society was prepared to pay £200 or more to avoid a UTI.

**Table 3:** Results of deterministic and probabilistic cost-effectiveness, cost-utility, and cost-benefit analyses

	Number of observations*	**Mean cost, £ (95% CI)**	**Mean incremental cost, £ (95% CI)**	**Mean effect (95% CI)**	**Mean incremental effect (95% CI)**†	**Incremental cost-effectiveness ratio, £**	**Probability that prophylaxis is cost-effective**				
							1	2	3	4	5
**(A) Cost-effectiveness analysis**											
No prophylaxis group	131/180	3496·73 (2585·87 to 4407·59)	..	2·5 (2·17 to 2·83)	..	..	0·501	0·399	0·235	0·086	0·008
Prophylaxis group	140/181	3615·44 (2309·43 to 4921·46)	118·72	1·30 (1·07 to 1·53)	−1·20	98·79	0·499	0·601	0·765	0·914	0·992
**(B) Cost-utility analysis**											
No prophylaxis group	131/93	3496·73 (2585·87 to 4407·59)	..	0·652 (0·622 to 0·682)	..	..	0·521	0·441	0·362	0·309	0·212
Prophylaxis group	140/96	3615·44 (2309·43 to 4921·46)	118·72	0·676 (0·643 to 0·708)	0·023	5059	0·479	0·559	0·638	0·691	0·788
**(C) Cost-benefit analysis**											
Adjusted analysis	202	..	−85·66 (−1943·33 to 1772·01)	..	208·72‡(1·49 to 415·94)	..	..	..	..	..	..

Data were not adjusted for missing data. (A) Data were calculated by use of the relative frequency of urinary tract infections from the primary outcome analysis. For the probability of cost-effectiveness, the pay threshold values for society's willingness to pay are 1: £0; 2: £200; 3: £500; 4: £1000; and 5: £2000. (B) Data were calculated by use of utility values from participant completion of the Medical Outcomes Short Form-36 questionnaire. For the probability of cost-effectiveness, the pay threshold values for society's willingness to pay are 1: £0, 2: £10 000, 3: £20 000, 4: £30 000, 5: £50 000. (C) Data were calculated by use of maximum willingness to pay to avoid one urinary tract infection and adjusted with the seemingly unrelated regression (sureg) function in STATA for estimation of costs and monetary benefits regressions.

* Data are cost/outcomes or all observations.

† Assessed by use of number of urinary tract infections, quality-adjusted life-years, and willingness to pay to avoid a urinary tract infection.

‡ Value that participants would be willing to pay to avoid a urinary tract infection multiplied by the number of urinary tract infections reported by participants (with the primary outcome definition).

19 (9%) participants in the prophylaxis group reported 22 non-serious adverse events due to antibiotic prophylaxis (table 4) and 13 (6%) of these 19 participants changed their originally selected agent or stopped prophylaxis during the 12-month trial. The incidence of adverse events with all three drugs used for prophylaxis was similar. At each of the 3-monthly reviews, less than 10% of participants in the prophylaxis group perceived adverse effects to be due to the use of prophylactic antibiotics. At the time of UTI, 28 (14%) of 203 participants in the prophylaxis group reported ever having adverse events due to treatment antibiotics versus 60 (30%) of 201 participants in the no prophylaxis group—predominately nausea (20 [10%] of 203 *vs* 38 [19%] of 201), diarrhoea (13 [6%] of 203 *vs* 31 [15%] of 201), and candida infection (ten [5%] of 203 *vs* 19 [10%] of 201). The low numbers of adverse events in each category precluded statistical analysis.

**Table 4:** Number of adverse events associated with prophylactic and treatment antibiotics

	**Prophylaxis group (n=203)**	**No prophylaxis (n=201)**
Prophylaxis antibiotics only*		
0 events	184 (91%)	197 (98%)
1 event	17 (8%)	3 (2%)
2 events	1 (<1%)	1 (<1%)
3 events	1 (<1%)	0
**Prophylaxis antibiotics only**†		
1 month	17 (8%)	0
3 months	20 (10%)	0
6 months	17 (8%)	0
9 months	10 (5%)	0
12 months	10 (5%)	2 (1%)
**Treatment antibiotics only**‡		
Any adverse event	28 (14%)	60 (30%)
Skin rash	2 (1%)	6 (3%)
Nausea	20 (10%)	38 (19%)
Diarrhoea (loose or more frequent bowel movement)	13 (6%)	31 (15%)
Thrush (candidal infection) in the mouth or vagina	10 (5%)	19 (10%)
Other antibiotic side-effects	4 (2%)	9 (5%)

* Data are the number of participants who reported adverse events in a health-care record review, completed by local trial research staff and assessed as being related to (or possibly related to) prophylaxis treatment.

† Data are the number of adverse events reported in each 3-monthly participant review, completed by local trial research staff and the participant.

‡ Data are the number of adverse events associated with treatment antibiotic ever reported in a urinary tract infection record form over the 12 months of trial participation, completed by the participant.

The conclusion of the primary analysis was unchanged when removing duration of antibiotic course for UTI treatment from the evaluation (IRR 0·50; 95% CI 0·43–0·58). This result was also unaffected by inclusion of the stratification factors (sex, previous frequency of UTIs, neurological bladder dysfunction) and other possible confounders (age, functional cause of poor bladder emptying, use of non-hydrophilic catheter, frequency of CISC, use of prophylaxis in previous 12 months, kidney function, and baseline bacteriuria) in the model. Sensitivity analyses for the primary outcome that used negative binomial regression with a strict intention-to-treat definition gave very similar results (negative binomial regression: IRR 0·52, 95% CI 0·42–0·65; p<0·0001; strict-intention-to-treat analysis: 0·53, 0·45–0·62; p<0·0001).

## Discussion

This trial provides clear, robust evidence of the effectiveness of continuous low-dose antibiotic prophylaxis as a preventive strategy against repeated UTIs for adults using CISC who have recurrent UTIs, which we showed over 12 months with three drugs that are licensed in the UK. The lower 95% confidence limit for the risk reduction (39%) in the primary outcome surpassed our prespecified minimal clinically important difference (20%). This confidence limit was also greater than that seen in a previous study^6^ of children. The benefits of prophylaxis were valued by participants in the prophylaxis group, who were satisfied with treatment and most of whom elected to continue prophylaxis long-term. However, the measured health status of participants was not improved from the impaired values at baseline in either group.

Increased development of antimicrobial resistance over the 12-month trial duration in the prophylaxis group is a major concern that limits the appeal of the prophylaxis strategy. This finding was particularly evident for drugs that were used for prophylaxis in the trial but was also seen for other antibiotics that are commonly prescribed for treatment of UTI. This finding was broadly consistent with a trial^7^ done in children.

This study was designed, conducted, analysed, and reported in accordance with best practice. We recruited participants from a diverse population of CISC users across a range of settings; however, our subsequent analyses found the effectiveness of prophylaxis to be consistent across clinical subgroups, which suggests generalisability across populations of adult users of CISC with similar characteristics in other countries, such as France and the Netherlands.^7^, ^17^ Regarding study design, use of a remote computerised randomisation system ensured concealment of allocation sequence; all likely confounders were balanced across the two groups and their inclusion in the statistical model did not affect the primary result; participants, treating clinicians, and local research staff could not be masked to allocation, but outcome assessors were masked; and we chose participant-reported UTIs as the primary outcome, with a definition that reflected participant experience in terms of UTI symptoms and actions by clinicians to provide a treatment course of an appropriate antibiotic. We also included different opportunities for participant reporting of UTIs and for research staff to confirm episodes at scheduled visits. An absence of participant blinding and reliance on patient report could have risked differential outcome reporting between groups. However, we believe that this possibility was unlikely because UTI reports were cross-checked against data from 3-monthly participant contact and validated against prespecified criteria. Similar findings between microbiologically confirmed UTIs and patient reports gave further reassurance that there was low detection bias. We did not assess the primary outcome in a small number of participants with less than 6 months' follow-up. The number of participants who were excluded from the primary analysis were balanced across groups, and a sensitivity analysis showed that inclusion of all randomised participants did not significantly affect these conclusions.

More frequent patient contact and education given during the trial around non-antibiotic interventions to reduce risk and the effects of UTIs could have decreased the number of episodes of UTIs that prompted patients to request antibiotics. It is also possible that we did not capture all symptomatic, antibiotic-treated UTI episodes, although numbers of missed events are likely to be similar across the two groups.

Our confidence in the trial findings is reinforced by the low number of withdrawals and high number of patients who completed follow-up. All prespecified sample size thresholds for the primary analysis were met. The proportion and type of missing data was also similar between groups. Further, to provide a comprehensive assessment of the effect on antimicrobial resistance, we included collection and centralised analysis of urine samples obtained during UTIs (representing urinary pathogens) and during asymptomatic periods (representing urinary colonisers) and perianal swabs (representing the faecal microbiome). All samples used in the antimicrobial resistance analyses were processed with consistent methods by a single, accredited laboratory and resistance was detected in accordance with European standards.

A detailed economic evaluation was done as part of the trial, triangulating three methods: cost-effectiveness, cost-utility, and cost-benefit. All three methods suggested that the benefit of antibiotic prophylaxis was potentially worthwhile, incurring a modest extra health-care cost. Quantification of the degree and duration of the detrimental effect of UTIs on an individual's wellbeing was problematic. We used a standard measure of health status; however, this did not show a significant decrease in health status during UTIs, and differences between groups over 12 months were small and unlikely to be socially significant. This difficulty measuring the effects of UTIs on health status was highlighted in a 2012 review.^18^ The small differences in the SF-36 score that we found were less than the minimal clinically important difference that was defined in a previous study.^19^ This elevation did not account for the potential cost of development of antimicrobial resistance. This limitation might make an important difference to estimates of cost-effectiveness but, unfortunately, precise estimates of economic effects are not available.^20^

The effectiveness of continuous low-dose antibiotic prophylaxis against UTIs in users of CISC who had repeated episodes increases the number of patient groups with repeated or chronic infections who have been shown to benefit from this strategy. The increased frequency of antimicrobial resistance in potential urinary pathogens to antibiotics that are used for treatment of UTIs is a concern for individuals and for public health. Increased resistance occurred in the prophylaxis group despite reduced use of antibiotics to treat symptomatic UTIs. The mechanism by which low-dose antibiotic prophylaxis provides a benefit is uncertain. Our results show that episodes of symptomatic UTIs (indicating infection with urinary pathogens) are suppressed while the incidence of asymptomatic bacteriuria (indicating urinary colonisation) was unchanged. It is possible that pathogenic bacterial strains with particular virulence factors are preferentially targeted by low-dose continuous therapy, or changes in the host response might be induced, allowing tolerance of colonisers but eradication of pathogens.^21^ Alternatively, bacteria colonising the bladder could be protected in specific niches that cannot be accessed by low urinary concentrations of antibiotic.

Evidence-based alternative ways of reducing the risk of UTIs and the severity of symptoms in women include non-antibiotic prophylaxis with methenamine hippurate^22^ and vaginal oestrogen supplementation; however, neither has been tested for CISC users.^23^ Frequency of use of non-antibiotic preventative strategies was similar in both groups of this trial: increased fluid intake, increased frequency of catheterisation, and ingestion of cranberry products and probiotics were the most popular interventions (data not shown). Cranberry extracts, although often taken, do not appear effective in reducing the risk of UTIs in CISC users.^24^, ^25^

An alternative antibiotic-based strategy is so-called self-start therapy, whereby patients have a supply of antibiotics at home and can start a treatment course when their typical symptoms occur.^26^ In the present trial, participants in the control group reported self-start therapy to be the source of their treatment antibiotics more often than the prophylaxis group, but we cannot speculate further from these data as to whether this treatment option is a viable alternative to continuous prophylaxis in our trial population.

Striking a balance between clear benefit to the individual by use of antibiotic prophylaxis and the potential for long-term individual and societal harm from higher frequency of antimicrobial resistance is difficult for patients and clinicians alike. In many countries, prescribers and health-care organisations are encouraged to follow the principles of antibiotic stewardship, which include avoidance of long-term use of antibiotics particularly at sub-bactericidal dosage.^27^ For CISC users of both sexes who have repeated UTIs, there do not appear to be any effective alternatives and the situation is further complicated by the high incidence of urinary bacterial colonisation (asymptomatic bacteriuria), which was unaffected by use of antibiotic prophylaxis; these findings are similar to those seen in children using CISC.^7^ It could be argued that our data show that the effect of repeated UTIs on patient wellbeing is low, showing few severe infections and little detriment to health status. However, a qualitative study nested within the trial revealed substantial distress that resulted from repeated UTI episodes among a relevant sample of participants,^28^ which is in agreement with a previous study^29^ of women seeking treatment for UTIs. Laboratory and clinical studies are required to understand better how continuous low-dose antibiotic prophylaxis works to find ways of preserving benefit but minimising the effect on antimicrobial resistance. This balance is likely to involve careful study of genotypic and phenotypic switching of urinary bacteria from non-invasive colonisers to symptom-causing pathogens and the effects of host response in these changes.

Our data will help clinicians worldwide to provide more accurate information on the benefits and harms of antibiotic prophylaxis in this patient group. These data will aid more balanced decision making for CISC users who have repeated UTIs, considering the degree of individual patient distress and emerging threats from antimicrobial resistance.

**This online publication has been corrected. The corrected version first appeared at thelancet.com/infection on August 22, 2018**
