# Corrections

**DOI:** 10.1016/S1473-3099(18)30504-8

**Published:** 2018-09

*Fisher H, Oluboyede Y, Chadwick T, et al. Continuous low dose antibiotic prophylaxis for adults performing clean intermittent catheterisation who suffer repeated urinary tract infection (AnTIC): a randomised, open-label, superiority trial.* Lancet Infect Dis *2018;* **18:** 957–68—In this Article, the corresponding author has changed to Dr Holly Fisher because of the death of Prof Robert Pickard on July 24, 2018. This correction has been made to the online version as of Aug 22, 2018, and the printed Article is correct.

