# Supplementary material for: Continuous low-dose antibiotic prophylaxis for adults with repeated urinary tract infections (AnTIC): a randomised, open-label trial
Source: Lancet Infect Dis. 2018 Sep;18(9):957–68. doi: 10.1016/S1473-3099(18)30279-2 (PMC6105581; doi:10.1016/S1473-3099(18)30279-2)
Supplement: Supplementary appendix [file mmc1.pdf]

THE LANCET
Infectious Diseases

Supplementary appendix

This appendix formed part of the original submission and has been peer reviewed. We post it as supplied by the authors.

Supplement to: Fisher H, Oluboyede Y, Chadwick T, et al. Continuous low-dose antibiotic prophylaxis for adults with repeated urinary tract infections (AnTIC): a randomised, open-label trial. *Lancet Infect Dis* 2018; published online June 28. http://dx.doi.org/10.1016/S1473-3099(18)30279-2.

Web Supplementary Material

Continuous low-dose antibiotic prophylaxis for adults with repeated urinary tract infections (AnTIC): a randomised, open-label trial

	Prophylaxis (included in primary analysis n = 181)	No prophylaxis (included in primary analysis n = 180)
**Age (years)**	59·3 (16·5)	59·9 (15·5)
**Weight (kg)**	78·0 (16·2)	81·4 (16·1)
**Stratification factors at randomisation**		
**Sex**		
Male	101 (55·8%)	104 (57·8%)
Female	80 (44·2%)	76 (42·2%)
**Number UTI episodes in 12 months prior to randomisation**		
<4	69 (38·1%)	68 (37·8%)
≥4	112 (61·9%)	112 (62·2%)
**Cause of bladder dysfunction**		
Neurological	71 (39·2%)	71 (39·4%)
Non-neurological	110 (60·8%)	109 (60·6%)
**Creatinine clearance (mL/min)**	89·2 (68·6, 119·8)	100·1 (74·5, 122·3)
**Type of intermittent catheterisation**		
By self	179 (98·9%)	177 (98·3%)
By spouse or carer	1 (0·6%)	2 (1·1%)
Missing	1 (0·6%)	1 (0·6%)
**Planned future duration of need for intermittent catheterisation**		
Between 1 and 2 years	0 (0·0%)	2 (1·1%)
Between 2 and 5 years	0 (0·0%)	1 (0·6%)
Indefinite	163 (90·1%)	164 (91·1%)
Not known	17 (9·4%)	12 (6·7%)
Missing	1 (0·6%)	1 (0·6%)
**Route of intermittent catheterisation**		
Urethra	174 (96·1%)	175 (97·2%)
Mitrofanoff catheterisable stoma	6 (3·3%)	4 (2·2%)
Missing	1 (0·6%)	1 (0·6%)
**Catheterisation details:**		
**Type of catheter used**		
Single use	174 (96·1%)	175 (97·2%)
Re-useable	6 (3·3%)	4 (2·2%)
Missing	1 (0·6%)	1 (0·6%)
**Hydrophilic coated catheter used?**		
No	7 (3·9%)	7 (3·9%)
Yes	169 (93·4%)	172 (95·6%)
Missing	5 (2·8%)	1 (0·6%)
**Frequency of CISC per 24 hours**	3·8 (2·2)	4·2 (3·0)
**Main functional reason for requiring intermittent catheterisation**		
Bladder outlet obstruction	45 (24·9%)	48 (26·7%)
Bladder failure (underactivity)	122 (67·4%)	117 (65·0%)
Bladder augmentation /replacement	12 (6·6%)	14 (7·8%)
Missing	2 (1·1%)	1 (0·6%)
**Urinary Infection:**		
**Episodes of UTI experienced by participant in 12 months prior to randomisation**	4·0 (3·0, 6·0)	4.0 (3·0, 7·0)
**Positive urine culture reports in 12 months prior to randomisation**	2·0 (1·0, 4·0)	2.0 (1·0, 4·0)
**Months use of antibiotic prophylaxis for UTI in 12 months prior to randomisation**	0·0 (0·0, 1·0)	0.0 (0·0, 1·0)
**Central laboratory culture of urine at baseline**		
Negative	81(44·8%)	74 (41·1%)
Positive	73 (40·2%)	72 (40·0%)
Missing	27 (14·9%)	34 (18·9%)

Supplementary Table 1: Baseline characteristics for participants included in the primary analysis only. Data are n (%), median [Inter quartile range (IQR)] or mean [standard deviation (SD)].

	Prophylaxis<br>n = 181<br>(included in primary analysis)	No prophylaxis<br>n = 180<br>(included in primary analysis)	IRR (95% CI)	p-value
**Symptomatic, antibiotic treated UTI (primary outcome)**				
Sex<br>Female<br>Prophylaxis group	n = 181	n = 180	 1.11 (0.95, 1.29)<br>0.50 (0.43, 0.58)	 0.18<br><0.0001
Previous frequency of UTI<br>≥4<br>Prophylaxis group	n = 181	n = 180	 1.94 (1.63, 2.31)<br>0.50 (0.42, 0.58)	 <0.0001<br><0.0001
Neurological bladder dysfunction<br>No<br>Prophylaxis group	n = 181	n = 180	 0.97 (0.83, 1.13)<br>0.50 (0.43, 0.58)	 0.68<br><0.0001
Age<br>Prophylaxis group	n = 181	n = 180	1.00 (1.00, 1.00)<br>0.52 (0.44, 0.61)	0.95<br><0.0001
Functional cause of poor bladder emptying (reference: Bladder outlet obstruction)<br>Bladder failure (underactivity)<br>Bladder augmentation/replacement<br>Prophylaxis group	n = 179	n = 179	 1.17 (0.98, 1.40)<br>1.39 (1.03, 1.87)<br>0.52 (0.44, 0.61)	 0.09<br>0.03<br><0.0001
Use of hydrophilic catheter<br>Yes<br>Prophylaxis group	n = 176	n = 179	 1.43 (0.90, 2.29)<br>0.52 (0.44, 0.61)	 0.13<br><0.0001
Frequency of CISC<br>Prophylaxis group	n = 181	n = 179	1.00 (0.97, 1.02)<br>0.52 (0.44, 0.61)	0.81<br><0.0001
Use of prophylaxis in previous 12 months<br>Prophylaxis group	n = 181	n = 180	1.03 (1.00, 1.05)<br>0.52 (0.44, 0.60)	0.08<br><0.0001
Kidney function at baseline (eGFR)<br>Prophylaxis group	n = 181	n = 176	1.00 (1.00, 1.00)<br>0.52 (0.44, 0.61)	0.14<br><0.0001
Baseline bacteriuria<br>Yes<br>Prophylaxis group	n = 149	n = 149	 0.92 (0.78, 1.09)<br>0.52 (0.44, 0.62)	 0.32<br><0.0001

Supplementary Table 2: Additional modelling of the primary outcome (incidence of symptomatic, antibiotic treated UTI). Each row displays the results of fitting a separate multivariate model including group and covariate.

	Prophylaxis<br>(n = 203)<br>(eligible n = 181)	No prophylaxis<br>(n = 201)<br>(eligible n = 180)		
	**Incidence rate**	**Incidence rate**	**IRR (95% CI)**	**p-value**
**Symptomatic, antibiotic treated UTI (primary outcome)**				
All eligible	1·3 (1·1, 1·6)	2·6 (2·3, 2·9)	0·52 (0·42, 0·65)	<0·0001
Subgroup: baseline episodes of UTI < 4	0·8 (0.6, 1·1)	1·7 (1·4, 2·2)	0·46 (0·31, 0·69)	0·52‡
Subgroup: baseline episodes of UTI ≥ 4	1·7 (1·3, 2·0)	3·1 (2·7, 3·6)	0·54 (0·42, 0·70)	

Supplementary Table 3: Sensitivity analysis for primary outcome (incidence of symptomatic antibiotic-treated UTI over 12 months) using <u>negative binomial regression.</u> ‡For interaction between subgroups (<4 and ≥4 infections at baseline) and treatment group.

	Prophylaxis<br>(n = 203)	No prophylaxis<br>(n = 201)		
	**Incidence rate**	**Incidence rate**	**IRR (95% CI)**	**p-value**
**Symptomatic, antibiotic treated UTI (primary outcome)**				
All eligible	1·4 (1·1, 1·6)	2·6 (2·3, 2·9)	0·53 (0·45, 0·62)	<0·0001
Subgroup: baseline episodes of UTI < 4	0·8 (0·6, 1·1)	1·7 (1·4, 2·2)	0·47 (0·34, 0·65)	0·43‡
Subgroup: baseline episodes of UTI ≥ 4	1·7 (1·3, 2·3)	3·1 (2·7, 3·6)	0·55 (0·46, 0·65)	

Supplementary Table 4: Sensitivity analysis for primary outcome (incidence of symptomatic antibiotic-treated UTI over 12 months using <u>strict intention-to-treat definition.</u> ‡For interaction between subgroups (<4 and ≥4 infections at baseline) and treatment group.

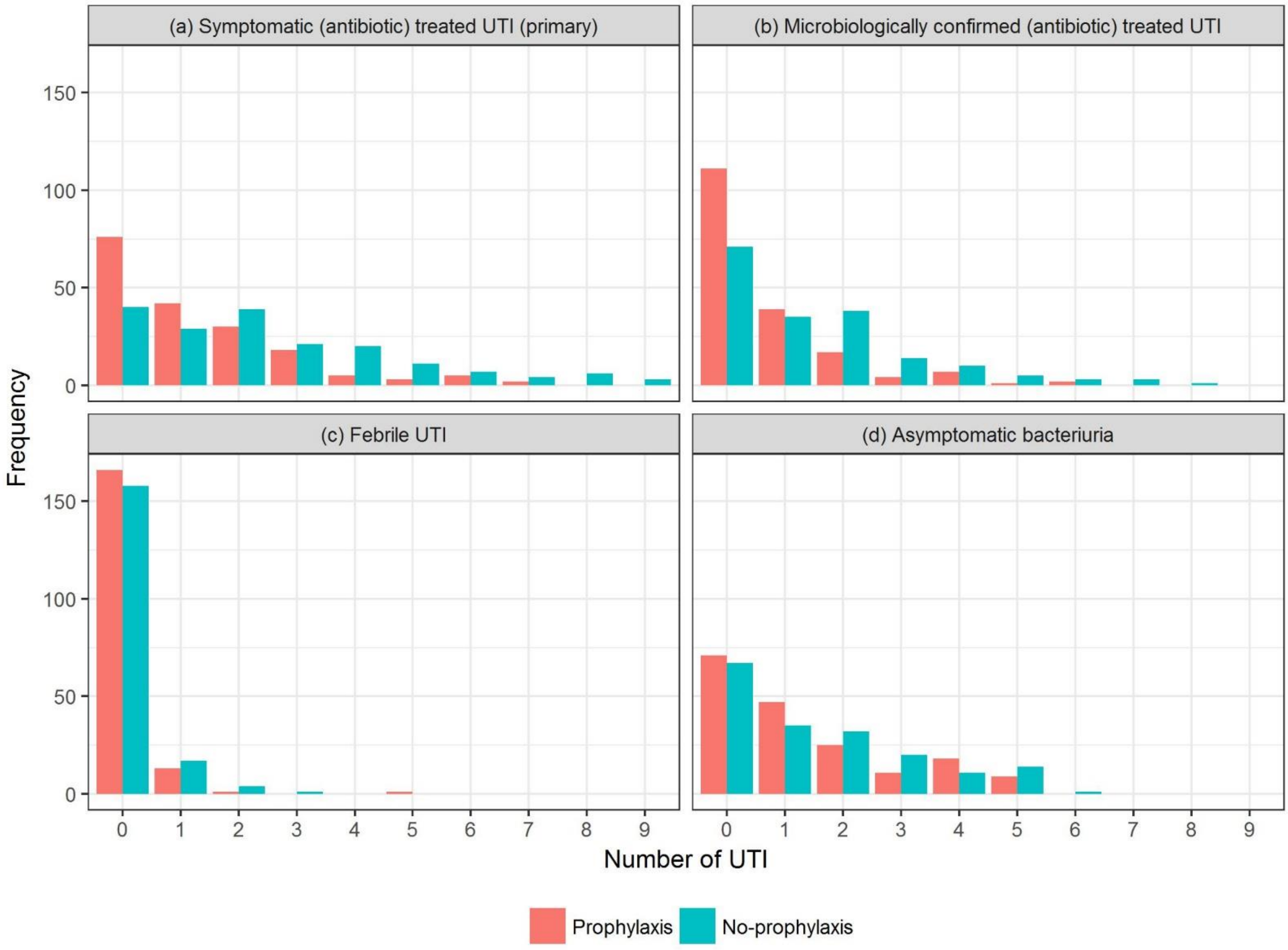

Supplementary Figure 1: Bar charts showing frequency distribution for occurrence of (a) primary outcome (symptomatic antibiotic-treated UTI) and secondary outcomes (b) microbiologically confirmed UTI, (c) febrile UTI and (d) asymptomatic bacteriuria) over 12 months of trial duration.

	Prophylaxis (n = 203)	No prophylaxis (n = 201)	Comparison between groups	
	mean (SD)	mean (SD)	t-test mean difference (95% CI), p-value	ANCOVA* p-value
**GFR**				
Baseline	86.6 (30.2) (n=200)	88.0 (26.1) (n=197)	-	-
12 months	82.3 (30.0) (n=123)	83.3 (27.6) (n=140)	-	-
Change from baseline	-2.1 (15.0) (n=123)	-3.4 (14.2) (n=138)	1.29 (-2.3, 4.9)<br>p = 0.48	p = 0.48
**ALT**				
Baseline	24.7 (19.9) (n=198)	24.1 (15.6) (n=188)	-	-
12 months	26.1 (19.1) (n=118)	24.0 (14.1) (n=135)	-	-
Change from baseline	1.2 (19.5) (n=117)	-0.3 (14.7) (n=128)	1.5 (-2.8, 5.8)<br>p = 0.49	p = 0.50

Supplementary Table 4: Mean [standard deviation (SD)] glomerular filtration rate (GFR) and alanine transaminase (ALT) at baseline and 12 months, with comparison between groups of change from baseline. *Adjusted for stratification factors at randomisation.

	Prophylaxis (n = 203)	No prophylaxis (n = 201)	Comparison between groups	
**Domain**	mean (SD)	mean (SD)	t-test mean difference (95% CI), p-value	ANCOVA* p-value
**Effectiveness**	78.0 (19.1) (n=144)	66.3 (19.5) (n=108)	11.7 (6.8, 16.5)<br>p < 0.001	p < 0.001
**Side-effects**	67.4 (23.4) (n=22)	67.2 (24.2) (n=32)	0.24 (-13.0, 13.5)<br>p = 0.97	p = 0.94
**Convenience**	88.9 (13.9) (n=144)	78.2 (21.4) (n=109)	10.7 (6.4, 15.1)<br>p < 0.001	p < 0.001
**Overall**	73.8 (25.4) (n=143)	63.0 (24.3) (n=109)	10.8 (4.5, 17.0)<br>p = 0.001	p < 0.001

Supplementary Table 5: Treatment satisfaction questionnaire for medication (TSQM) scores at 12 months, with comparisons by group (maximum rating = 100 for each domain). *Adjusted for stratification factors used at randomisation

	Nitrofurantoin (n = 71)	Trimethoprim (n = 92)	Cefalexin (n =33)
**Number of participants who reported adverse events in a health-care record review, completed by local trial research staff and assessed as being related to (or possibly related to) prophylaxis treatment**			
0 events	62 (87%)	85 (92%)	30 (91%)
1 events	8 (11%)	6 (7%)	3 (9%)
2 events	0 (0%)	1 (1%)	0 (0%)
3 events	1 (1%)	0 (0%)	0 (0%)
**Number of participants who reported adverse events in each 3-monthly participant review, completed by local trial research staff and the participant**			
1 month	6 (8%)	9 (10%)	2 (6%)
3 month	9 (13%)	10 (11%)	1 (3%)
6 month	9 (13%)	7 (8%)	1 (3%)
9 month	3 (4%)	4 (4%)	2 (6%)
12 month	3 (4%)	6 (7%)	1 (3%)

Supplementary Table 6: Number of adverse events associated with prophylactic antibiotic by initial choice of agent, prophylaxis group only, n = 196 (7 participants in the prophylaxis group did not receive prophylaxis).

	Initial agent (n = 196)	Switched agent ever	Switched to nitrofurantoin	Switched to trimethoprim	Switched to cefalexin
**Initial agent:**					
Nitrofurantoin	71 (36%)	8	-	6	2
Trimethoprim	92 (47%)	15	7	-	8
Cefalexin	33 (17%)	2	1	1	-

Supplementary Table 7: Choice of initial agent and agent switched to during trial (no participants switched more than once), prophylaxis group only, n = 196 (7 participants in the prophylaxis group did not receive prophylaxis).

Trial allocated group		Prophylaxis (n = 203)	No prophylaxis (n = 201)
Management strategy in use at 18 months	Prophylaxis	72 (35.5%)	18 (9.0%)
	No prophylaxis	36 (17.7%)	72 (35.8%)
	Missing	95 (46.8%)	111 (55.2%)
Urine culture during asymptomatic state at 18 months	Positive	61 (30.1%)	35 (17.4%)
	Negative	23 (11.3%)	29 (14.4%)
	No sample received	119 (58.6%)	137 (68.2%)
Escherichia coli isolated from perianal swab at 18 months	Positive	27 (13.3%)	15 (7.5%)
	Negative	44 (21.7%)	36 (17.9%)
	No sample received	132 (65.0%)	150 (74.6%)

Supplementary Table 8: Participants submitting data for the optional additional microbiological follow up at 18 months (six months after completion of 12-month trial period) by trial allocated group

	n (%)	Mean (SD)	Median
**Baseline**			
**Prophylaxis (n = 203)**			
SF-36 MCS	194 (95.6)	48.68 (12.22)	51.68
SF-36 PCS	193 (95.1)	41.02 (12.12)	41.61
**No Prophylaxis (n = 201)**			
SF-36 MCS	187 (93.0)	49.39 (12.05)	52.84
SF-36 PCS	186 (92.5)	40.13 (11.98)	40.58
**6 months**			
**Prophylaxis (n = 203)**			
SF-36 MCS	148 (72.9)	49.2 (12.55)	53.93
SF-36 PCS	146 (71.9)	39.85 (12.36)	39.66
**No Prophylaxis (n = 201)**			
SF-36 MCS	147 (73.1)	46.24 (12.85)	49.47
SF-36 PCS	144 (71.6)	39.47 (12.15)	39.23
**12 months**			
**Prophylaxis (n = 203)**			
SF-36 MCS	139 (68.5)	48.06 (12.39)	53.03
SF-36 PCS	137 (67.5)	39.43 (13.07)	38.42
**No Prophylaxis (n = 201)**			
SF-36 MCS	137(68.2)	46.99 (13.08)	51.75
SF-36 PCS	134 (66.7)	39.83 (11.95)	39.74

Supplementary Table 9: Mean, standard deviation (SD) and median for SF-36 mental component score (MCS) and physical component score (PCS) by study group at each time point.

	Baseline	6 months	12 months	At time of UTI	Area under the curve for 0-12 months unadjusted	Area under the curve for 0-12 months adjusted for utility value at time UTI
**All participants (n = 404)**	0.69 (0.16), n = 391	0.66 (0.16), n=319	0.64 (0.17), n=296	0.63 (0.19), n=314	0.66 (0.16), n=289	0.65 (0.19), n=283
**Prophylaxis (n=203)**	0.69 (0.17), n=197	0.67 (0.17), n=164	0.64 (0.18), n=150	0.64 (0.17), n=159	0.68 (0.16), n=147	0.64 (0.20), n=147
**No prophylaxis (n=201)**	0.69 (0.14), n=194	0.65 (0.16), n=155	0.64 (0.15), n=146	0.62 (0.20), n=155	0.65 (0.15), n=142	0.65 (0.17), n=136

Supplementary Table 10: Mean [standard deviation (SD)] utility values derived from participant completion of the SF-36 at baseline, 6 and 12 months and at time of symptomatic UTI by study group.

	Prophylaxis				No prophylaxis			
	**Users**	**Mean**	**Median**	**SD**	**Users**	**Mean**	**Median**	**SD**
Inpatient days in hospital	24	19.42	10.00	24.22	36	12.72	4.00	17.38
Day Case admissions	38	13.53	2.00	41.83	42	5.29	2.00	6.87
Outpatient visits	103	14.83	6.00	27.88	94	14.13	8.00	22.74
A&E (accident and emergency) visits	21	11.14	2.00	37.84	18	2.44	2.00	1.76
GP Surgery consultations	113	13.22	8.00	25.22	120	10.05	8.00	7.65
GP Home consultations	12	3.33	4.00	2.15	12	6.17	2.00	7.46
Nurse Surgery consultations	94	8.66	4.00	21.46	83	10.36	4.00	30.58
Nurse Home consultations	20	33.90	4.00	64.02	14	40.57	5.00	94.62
GP telephone consultations	43	5.21	4.00	4.68	45	6.36	4.00	9.34
Hospital doctor telephone consultations	7	3.71	2.00	2.43	7	3.71	2.00	2.14
Nurse telephone consultations	26	5.54	3.00	7.38	27	5.04	4.00	5.56
Telephone consultations with other health care professionals	13	12.15	4.00	29.51	16	4.50	4.00	3.14
GP out of hours consultations	6	4.00	3.00	2.53	5	3.20	2.00	2.68
Hospital doctor out of hours consultations	1	4.00	4.00	NA	4	1.50	1.00	1.91
Nurse out of hours consultations	2	2.00	2.00	0.00	8	4.50	2.00	5.42
Out of hour consultations with other clinicians	1	12.00	12.00	NA	5	1.60	2.00	1.67

Supplementary Table 11: Resource use over 12 months follow-up for those with full economic data

Resource	Total cost (£) across the two arms - Baseline to 6 months						Total cost (£) across the two arms – 6 months to 12 months					
	Prophylaxis			No Prophylaxis			Prophylaxis			No Prophylaxis		
	Mean	Median	Standard Deviation	Mean	Median	Standard Deviation	Mean	Median	Standard Deviation	Mean	Median	Standard Deviation
Inpatient days in hospital	5195.41	2301.20	4867.04	3497.14	2301.20	3119.96	4345.30	2301.20	4998.95	3561.53	1585.60	4857.78
Day Case admissions	1100.00	704.00	1177.14	1944.38	704.00	2191.52	6281.85	704.00	17603.86	1243.73	704.00	1391.93
Outpatient visits	633.27	420.36	773.37	791.09	420.36	869.46	1270.63	630.54	2967.08	1019.76	630.54	2339.55
A and E visits	411.21	293.72	151.68	326.36	293.72	97.91	2327.17	293.72	7071.13	293.72	293.72	177.12
GP Surgery consultations	188.61	144.00	148.61	221.25	144.00	163.60	364.32	180.00	940.87	215.30	216.00	136.20
GP Home consultations	148.20	88.92	102.68	160.06	177.84	74.40	133.38	177.84	86.41	248.98	88.92	277.42
Nurse Surgery consultations	58.91	43.20	81.40	40.72	21.60	28.19	76.04	43.20	247.49	115.32	43.20	384.05
Nurse Home consultations	280.00	72.00	551.51	486.00	72.00	1032.08	744.92	144.00	1281.96	609.00	54.00	1275.70
GP telephone consultations	53.84	28.80	36.12	53.88	28.80	39.87	68.52	57.60	59.83	90.67	57.60	132.39
Hospital doctor telephone consultations	28.80	28.80	NA	43.20	28.80	28.80	57.60	43.20	36.43	67.20	86.40	33.26
Nurse telephone consultations	18.51	12.96	11.17	14.10	8.64	9.64	24.19	8.64	31.19	24.59	17.28	22.54
Telephone consultations with other health care professionals	44.00	48.00	18.07	39.27	48.00	26.88	204.00	36.00	451.74	54.00	60.00	37.95
GP out of hours consultations	178.88	134.16	77.46	134.16	134.16	0.00	214.66	134.16	120.00	268.32	134.16	232.37
Hospital doctor out of hours consultations	0.00	0.00	NA	210.18	210.18	210.18	420.36	420.36	NA	0.00	0.00	NA
Nurse out of hours consultations	21.60	21.60	NA	10.80	10.80	15.27	21.60	21.60	NA	61.20	43.20	63.22
Out of hour consultations with other health care professionals	0.00	0.00	NA	210.18	210.18	210.18	1261.08	1261.08	NA	105.09	105.09	148.62
Out of pocket expenses for private health care	810.46	810.46	863.32	712.00	340.00	895.63	1387.99	370.16	2465.22	1185.71	280.00	1892.70

Supplementary Table 12: Total costs over 12 months follow-up including all participants with full economic data

Outcome measure and time point	Prophylaxis (included in primary analysis n = 181)			No prophylaxis (included in primary analysis n = 180)		
	N	Mean	Std. Deviation	N	Mean	Std. Deviation
**SF-6D at baseline**	178	0.668	0.168	178	0.663	0.145
**SF-6D at 6 months**	137	0.659	0.168	139	0.644	0.152
**SF-6D at 12 month**	127	0.649	0.182	122	0.643	0.155
**QALYs from baseline to 6 months**	121	0.335	0.079	124	0.329	0.069
**QALY from 6 to 12 months**	108	0.333	0.085	104	0.323	0.075
**QALY from baseline to 12 month**	96	0.676	0.162	93	0.652	0.147
**QALY from baseline to 6 months and utility impact of UTI and no multiple imputation**	130	0.302	0.137	125	0.301	0.127
**QALY from 6 to 12 months and utility impact of UTI and no multiple imputation**	95	0.333	0.088	78	0.329	0.076
**QALY from baseline to 6 months and utility impact of UTI and no multiple imputation**	93	0.641	0.205	74	0.650	0.170

Supplementary Table 13: Comparisons of outcomes Baseline, 6 and 12 months

Data response rates	Prophylaxis (included in primary analysis n = 181)	No prophylaxis (included in primary analysis n = 180)
**Health utilisation**		
**6 months**	157	152
**12 months**	146	152
**Complete data 6 & 12 months**	140	131
**SF-36**		
**Baseline**	178	178
**6 months**	137	139
**12 months**	127	122
**Complete data Baseline & 6 & 12 months**	96	93
**WTP**		
**13 months**	101	97

Supplementary Table 14: Response rates for economic data
